# Thickness Influence on *In Vitro* Biocompatibility of Titanium Nitride Thin Films Synthesized by Pulsed Laser Deposition

**DOI:** 10.3390/ma9010038

**Published:** 2016-01-13

**Authors:** Liviu Duta, George E. Stan, Adrian C. Popa, Marius A. Husanu, Sorin Moga, Marcela Socol, Irina Zgura, Florin Miculescu, Iuliana Urzica, Andrei C. Popescu, Ion N. Mihailescu

**Affiliations:** 1 National Institute for Lasers, Plasma and Radiation Physics, Magurele-Ilfov 077125, Romania; liviu.duta@inflpr.ro (L.D.); iiul@yahoo.com (I.U.); 2 National Institute of Materials Physics, Magurele-Ilfov 077125, Romania; george_stan@infim.ro (G.E.S.); ahusanu@infim.ro (M.A.H.); marcela.socol@infim.ro (M.S.); irina.zgura@infim.ro (I.Z.); 3 Army Centre for Medical Research, Bucharest 020012, Romania; adrian.claudiu@gmail.com; 4 Research Centre for Advanced Materials, University of Pitesti, Pitesti 110040, Romania; sorin.g.moga@gmail.com; 5 Faculty of Materials Science and Engineering, Politehnica University of Bucharest, Bucharest 060042, Romania; f_miculescu@yahoo.com

**Keywords:** titanium nitride, medical instrument, pull-out adherence, cytocompatibility, pulsed laser deposition

## Abstract

We report a study on the biocompatibility *vs.* thickness in the case of titanium nitride (TiN) films synthesized on 410 medical grade stainless steel substrates by pulsed laser deposition. The films were grown in a nitrogen atmosphere, and their *in vitro* cytotoxicity was assessed according to ISO 10993-5 [1]. Extensive physical-chemical analyses have been carried out on the deposited structures with various thicknesses in order to explain the differences in biological behavior: profilometry, scanning electron microscopy, atomic force microscopy, X-ray photoelectron spectroscopy (XPS), X-ray diffraction and surface energy measurements. XPS revealed the presence of titanium oxynitride beside TiN in amounts that vary with the film thickness. The cytocompatibility of films seems to be influenced by their TiN surface content. The thinner films seem to be more suitable for medical applications, due to the combined high values of bonding strength and superior cytocompatibility.

## 1. Introduction

The improvement in the performance and durability of tissue- and bone-cutting devices is dependent on the structural, morphological, mechanical and biological properties of hard coatings applied on the surface.

Titanium nitride (TiN) is a well-known, extremely hard, biocompatible ceramic material [2]; moreover, it possesses high electron conductivity and mobility, as well as a high melting point [3]. Its excellent physical and chemical properties, which can be varied in a broad range [4,5], have generated great interest and have been exploited in the field of hard and protective coatings [6,7,8,9]. We note that TiN presents good wear and corrosion resistance properties when used in physiological environments [10,11].

TiN films synthesized by ion beam deposition [12], direct current magnetron sputtering [13], cathodic arc evaporation [14], pulsed laser deposition [15,16], chemical precursor synthesis [17], chemical vapor deposition (CVD) and plasma-assisted CVD techniques [18,19] are used for a wide variety of applications, including biomedical ones [20,21], with specific demands for improving the wear resistance, adhesion to the substrate and fatigue [22]. After applying TiN coatings, the biocompatibility of implants manufactured from various metallic alloys (such as cobalt-chromium, chromium-nickel or titanium alloys) is improved, increasing the wear and corrosion resistance and avoiding allergic reactions that may occur when a metallic implant is introduced inside the human body [23,24].

Stainless steel is a versatile class of material that has high strength and resistance to oxidation. As compared to Ti, it is easy to machine and, thus, commonly used for surgical instruments, bone screws and other medical equipment [25]. Grade 410 stainless steel is the basic martensitic stainless steel. The surgical devices fabricated from martensitic stainless steel are currently used as a standard tool for soft and hard tissue surgery [26].

In the field of high-quality thin film growth, pulsed laser deposition (PLD) has proven to be a quite versatile method [27,28]. In this technique, a very intense pulsed laser beam is focused onto a target in order to ablate its surface under vacuum or different process gas atmospheres, and the vapors are collected on substrates in the form of thin films [27]. One common modification to the basic PLD technique involves the introduction of ambient gases during the deposition process [29]. In particular, nitrogen (N_2_) presence in the deposition chamber has the role of varying the chemical composition of the films [27]. This way, the properties of the coatings can be easily tailored for a wide variety of applications. The interaction of the plume with the environment, which takes place in the gas phase, but also on the target and substrate surface, plays an important role in generating the atomic and molecular precursors necessary for the growth of compound phases.

It is to be mention that currently, blade edges are covered with TiN films by different physical vapor deposition processes with the aim of obtaining cutting devices with important advantages, such as increased tool life and cutting speed rates [30].

The aim of the present study was to tailor the thickness of TiN films grown by the PLD method in order to be applied as thin protective layers for medical devices (e.g., drills, burrs, scalpels or chisels) used for cutting purposes in soft and hard tissue surgery. We shall show that even small differences in films thickness affect the biological response of these surfaces. A thorough physical-chemical investigation was conducted in order to unveil the factors that vary with thickness, and that are responsible for the particular biological behavior of films.

## 2. Experimental Section

### 2.1. PLD Experiment

PLD experiments have been performed inside a stainless steel reaction chamber using a KrF* excimer laser source (λ = 248 nm, τ_FWHM_ ≤ 25 ns), running at a repetition rate of 10 Hz. The laser beam was incident at 45° with respect to the target surface.

TiN pellets from Plasmaterials (2.5 cm diameter × 0.6 cm thickness) were used as targets, while grade 410-L stainless steel plates (further denoted as 410SS), with dimensions of 2.3 × 1.8 × 0.2 cm^3^, or Si (100) wafers and soda-lime glasses of 1 × 1 cm^2^ from Thermo Scientific were used as deposition substrates. A study was performed prior to the deposition experiments, taking all cautions to assure similar characteristics for the films, irrespective of the nature of the substrates. We note that all of the substrates had a mirror-polished surface quality. The experimental conditions were identical for all substrates. The target and substrate were assembled in a frontal (on-axis) geometry, at a 5-cm separation distance. The laser fluence onto the target surface was set at ~5 J/cm^2^ (the corresponding pulse energy was 500 mJ).

Before deposition, the targets were submitted to a “cleaning” procedure with laser pulses. During this treatment, a shutter was interposed between the target and the substrate to collect the potential remnant impurities and defects on the target surface. The targets were continuously rotated with 0.4 Hz and translated along two orthogonal axes to avoid drilling and to ensure a uniform deposition.

Prior to introduction inside the deposition chamber, in order to eliminate micro-impurities, the substrates were successively cleaned in an ultrasonic bath in acetone, ethanol and deionized water for 20 min and then blown dry with high purity N_2_. During deposition, the substrates were heated and maintained at a constant temperature of 500 °C using a PID-EXCEL temperature controller. A ramp of 25 °C/min was chosen for heating the substrates to reach the deposition temperature. Once the film growth was completed, cooling down to room temperature (RT) was performed in the same ambient gas atmosphere as used for the film growth, with a ramp of 10 °C/min.

Before each experiment, the reaction chamber was evacuated with a high vacuum installation down to a residual pressure of 10^−5^ Pa. The dynamic ambient gas pressure during the thin film growth was kept at around 0.2 Pa by feeding high-purity N_2_ into the chamber with the aid of a calibrated gas inlet. An MKS 4000 controller was used for monitoring the gas pressure.

Three sets of TiN samples with 5000 (further denoted as 5A), 10,000 (further denoted as 10B) and 20,000 (further denoted as 20C) subsequent laser pulses were deposited. The number of pulses corresponds to the following deposition times: 8 min and 20 s (5A), 16 min and 40 s (10B) and 33 min and 20 s (20C).

### 2.2. Physical-Chemical Characterization of Deposited Structures

#### 2.2.1. Biological Assays

##### 2.2.1.1. Cell Cultures

Fibroblasts, Hs27 cell-line from ATCC, were used to assess the cytotoxicity of TiN thin films. Cells were grown with Dulbecco’s Modified Eagle’s Medium with l-glutamine (DMEM), supplemented with 10% bovine fetal serum, penicillin (100 UI/mL) and streptomycin (100 µg/mL), in a humidified atmosphere incubator with 5% CO_2_, at 37 °C.

The square substrates with a surface of 1 cm^2^ were cleaned with 70% and absolute ethanol, sterilized with dry heat (180 °C/1 h) and transferred in sterile 24-well plates, in the cell culture laminar flow hood.

When cells reached confluence, they were detached with trypsin, collected and centrifuged at 250× *g* for 10 min after trypsin inhibition. Cells were re-suspended with complete growing medium, counted with a Bürker-Türk counting chamber, and the concentration was adjusted to 105 cells/mL. On each sample, 104 cells in 100 µL DMEM were seeded. The plates were put in the humidified atmosphere incubator and allowed to adhere for 5 h. After this interval, 400 µL were added, and the cells were allowed to grow in the incubator for another 24 h. After 24 h, the medium was collected in order to assess the cytotoxic effect with a lactate dehydrogenase (LDH) experiment, and cells were subjected to further analysis. All cell culture reagents were purchased from Sigma Aldrich (St. Louis, MO, USA).

##### 2.2.1.2. Cell Morphology

Cells grown on different samples (410SS bare substrate and 5A, 10B and 20C TiN films) were examined by means of fluorescence imaging to observe any morphology modifications that can occur after their development on the films. Cells were fixed with 4% para-formaldehyde dissolved in phosphate-buffered saline (PBS) for 15 min at RT. The cells were then washed thrice with PBS and then incubated at RT, for 1 h, with 100 µL of phalloidin-AlexaFluor546 (Invitrogen, Carlsbad, CA, USA) diluted accordingly to the manufacturer's specifications. Samples were washed thrice with PBS for 15 min each and then incubated with 1 µg/mL 4',6-diamidino-2-phenylindole (DAPI), produced by Sigma Aldrich (St. Louis, MO, USA). After incubation, the cells were washed twice with PBS and once with double-distilled water for 15 min (each wash) and then mounted using 0.17-mm thin glass microscopy coverslips and fluorescence mounting medium (Invitrogen). Samples were examined using a Zeiss Axioplan fluorescence microscope with appropriate filters.

##### 2.2.1.3. Proliferation Assay

Cells’ proliferation on the surface of the samples was investigated using an MTS kit (Promega, Madison, WI, USA). Briefly, after the cell culture medium was removed, 400 µL of fresh DMEM without phenol red were added, and plates were transferred it the incubator. After 30 min, in each well, 80 µL MTS ready-to-use reagent were added, and the plates were introduced back into the incubator. After 1 h of incubation, the plates were put on a gentle shaker (150 rpm/1 min), and then, 120 µL of medium were harvested from each well and transferred into 96 microplates. Absorption was read at 490 nm with a Zenyth 3100 multimode microplate reader (Anthos).

##### 2.2.1.4. Cell Toxicity Assay

Cell toxicity was investigated using an LDH activity Kit (Thermo Scientific, Waltham, MA, USA). After 24 h, the supernatant medium was harvested, and 50 µL were transferred in 96 micro-well plates. Fifty microliters of LDH substrate solution, prepared according to the producer’s specification, were added, and the plates were transferred to the incubator. After 30 min, the reaction was stopped by the addition of 50 µL of stop buffer, and absorptions were read at 490 and 620 nm with a Zenyth 3100 multimode microplate reader (Anthos). For each situation, the values were calculated by subtracting the 620-nm values from the 490 ones. The control of the experiment for LDH activity was represented by the fresh complete medium.

#### 2.2.2. Profilometry

The thickness of the TiN thin films was determined by profilometry using an Ambios Technology XP-2 Stylus Profiler (Santa Cruz, CA, USA). The profilometry measurements were performed at RT, on films deposited onto glass substrates.

#### 2.2.3. Scanning Electron Microscopy

The general morphology of the deposited films was investigated by scanning electron microscopy (SEM) with an FEI Inspect S electron microscope. The measurements were carried out at 5-kV acceleration voltages, in high vacuum, under secondary electron acquisition mode. Cross-section SEM images were recorded on samples deposited on Si (100) wafers, in order to evaluate film thickness.

#### 2.2.4. Atomic Force Microscopy

Further, more insightful morphology analyses were carried out by atomic force microscopy (AFM) with a MultiView 4000 Nanonics system (Jerusalem, Israel) working in phase feedback. For AFM investigations, glass substrates were used. For each TiN-deposited film, the root mean square (*R*_RMS_) and average (*R*_a_) roughness values were inferred. In order to check the uniformity of films, different zones (having areas of 2 × 2 μm^2^) on the same sample were scanned, and the corresponding images were acquired. All measurements were performed at RT.

#### 2.2.5. X-ray Photoelectron Spectroscopy

X-ray photoelectron spectroscopy (XPS) analyses were performed in a dedicated chamber (Specs GmbH, Berlin, Germany), under ultra-high vacuum (base pressure ~10^−9^ mbar), using Al *K*_α_ = 1486.71 eV monochromatized radiation. The electrons were collected using a hemispherical electron energy analyzer (Phoibos 150) operated at a 20-eV pass energy. Resolution (in terms of full width at half maximum) of 0.45 eV was achieved. During measurements, a flood gun operating at a 1-eV acceleration energy and a 1-mA electron current was used in order to ensure sample neutralization. The TiN samples were investigated in (i) normal incidence (N.E.) with respect to the electron analyzer and (ii) at a 60° take-off angle (T.O.) in order to increase the surface sensitivity. 

The experimental data were fitted with Voigt profiles (*i.e.*, a Gaussian line convoluted with a Lorentzian one). The Gaussian components account mainly for the instrumental resolution, while the Lorentzian line is connected to the finite core-hole lifetime associated with the photoionization process.

#### 2.2.6. X-ray Diffraction

The crystalline phase identification of films was carried out by X-ray diffraction (XRD) using a Bruker AXS D8 Advance diffractometer (Karlsruhe, Germany), with CuK_α_ radiation, with parallel beam optics in both symmetric (θ–θ) and grazing incidence (α = 2°) geometries. XRD patterns were recorded in the 2θ = 30°–80° angular range, with a step size of 0.04° and 25 s per step.

#### 2.2.7. Wetting

The wetting properties of the samples were studied by measuring the static contact angle (CA) with a Drop Shape Analysis system, Model DSA100, from Kruss GmbH (Hamburg, Germany). The samples (glass and 410SS) were placed on a planar stage, under the tip of a water-dispensing disposable blunt-end stainless steel needle with an outer diameter of 0.05 cm. For CA measurements, two water droplets were poured on each sample. The volume of one water droplet was approximately 2 μL. The needle was attached to a syringe pump controlled by a PC for the delivery of the water droplet to the test surface. The drop size and the drip distance were kept constant in all cases. The dispensing of the droplet and analysis of the CA and of other drop parameters were carried out by the PC using the DSA3^®^ software supplied with the instrument. CA was measured by fitting a polynomial equation of second degree or a circle equation to the shape of the sessile drop and then calculating the slope of the tangent to the drop at the liquid-solid-vapor interface line. The camera was positioned so as to observe the droplet under an angle of about 2°–3° with respect to the plane of the sample surface supporting the droplet. The tests were carried out at RT [31,32,33,34,35].

Solid surface free energy (SFE) calculations were performed based on CA measurements, using ethylene glycol and deionized water as standard wetting solvents. The concept of polar and dispersion components (Owens-Wendt approximation) was applied [36]. For each investigated surface, five experiments were carried out. The mean value and standard deviation (SD) were computed.

#### 2.2.8. Pull-Out

The adherence at the 410SS substrate-TiN films’ interface was measured by the pull-out method. This is a quantitative test that uses a certified adhesion tester, Model PAT MICRO AT101, from DFD Instruments (Kristiansand, Norway), equipped with 0.28-cm diameter stainless steel test elements. They were affixed onto the coating’s surface by a cyanoacrylate one-component epoxy adhesive, E1100S. The stub surface was first polished, ultrasonically degreased in acetone and ethanol and then dried in N_2_ flow. After gluing, the samples were placed in an oven for thermal curing (130 °C/1 h). By use of a portable pull-out adherence tester, a load was increasingly applied to the surface until the dolly was pulled off. Failure will occur along the weakest plane within the system comprised of the dolly, adhesive, coating system and substrate. The experimental procedure was conducted in accordance with the ASTM D4541 [37] and ISO 4624 [38] standards. Batches of five samples have been tested for each type of material, and a statistical estimation is given.

#### 2.2.9. Statistical Analysis

All experiments were carried out in triplicate in order to achieve statistical significance. The statistical analyses were performed using the unpaired Student’s *t*-test, and the differences were considered significant when *p* < 0.05.

## 3. Results

### 3.1. Thickness and Bonding Strength of TiN Films Deposited on 410SS Substrates

**Figure 1:**
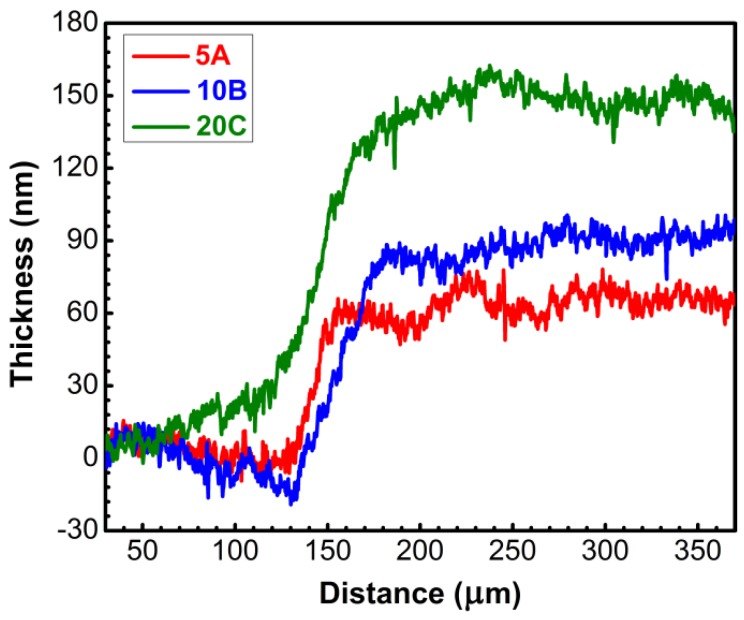
Thickness profiles of TiN layers recorded by profilometry.

The thicknesses of TiN films deposited on soda-lime glass substrates, as determined by profilometry, are collected in Table 1. We note that the recorded values include the presence (if any) of an interfacial layer, which could be the result of inter-diffusion in the first stages of film deposition. The experimental profiles are presented in Figure 1. As expected, a progressive increase of the film thickness is obtained with the increase of the number of laser pulses applied on the target.

**Table 1:** Thickness of TiN layers as determined by profilometry measurements.

Sample Type	Film Thickness (nm)
5A film	60.6 ± 11.7
10B film	86.6 ± 13.8
20C film	133.1 ± 18.2

The mean bonding strength values of TiN-coated 410SS obtained by the pull-out test are collected in Table 2. We note that the films have been clean detached, as visible by the naked eye. The films bonding strength failure was of the adhesive type, the fracture always developing at the coating-substrate interface at values of ~55 MPa. No statistically-significant differences were recorded between samples (*p* > 0.05). The high values of adherence obtained for the TiN thin films should be emphasized, as this ensures their sustainability as biofunctional coatings of medical instruments.

**Table 2:** Mean bonding strength values of TiN films deposited on 410-L stainless steel (410SS) substrates.

Sample Type	Bonding Strength (MPa)
Control (bare 410SS)	~65
5A film	55.7 ± 3.9
10B film	57.5 ± 7.5
20C film	55.0 ± 7.1

### 3.2. Biological Assessment of TiN Films

Human fibroblasts (*i.e.*, Hs27 cell line) were used in our study to gauge the cytotoxicity of the as-deposited TiN films. The choice is justified by: (1) the reliability of this type of cell; and (2) the envisaged application of the coatings, *i.e.*, functionalization of medical instruments (e.g., drills, burrs, or chisels) used for hard tissue dislocation and which are subjected to numerous heat sterilizations during their lifetime. In the eventuality of film delamination during the medical procedure, some microscopic fragments of the film can remain in the living organism, and one must ensure that they have a good cytotoxicity in order to not impede the healing of the tissue. Fibroblasts are the chief cells implicated in wound healing, and thereby, it was considered of great relevance to use this type of cell for the *in vitro* assays.

As presented in Section 2.2.1.1, the cells were seeded on the 1-cm^2^ samples and allowed to proliferate for 24 h. After this period, the cell morphology, proliferation and death for the different types of coated and uncoated surfaces were investigated.

#### 3.2.1. Cell Morphology

The bare 410SS samples and the TiN films are opaque to light, and therefore, the optimal technique to examine the morphology of the cells was considered epi-fluorescence. This is a technique where the surface of the sample is illuminated with a specific wavelength, and the excited fluorophores, which stain certain structures of the cells, emit light with a longer wavelength that is observed by the examiner. Cells were stained with phalloidin-AlexaFluor546 to reveal the microfilaments of actin in the cells cytoskeleton and with DAPI to evidence the DNA in the cell nucleus.

**Figure 2:**
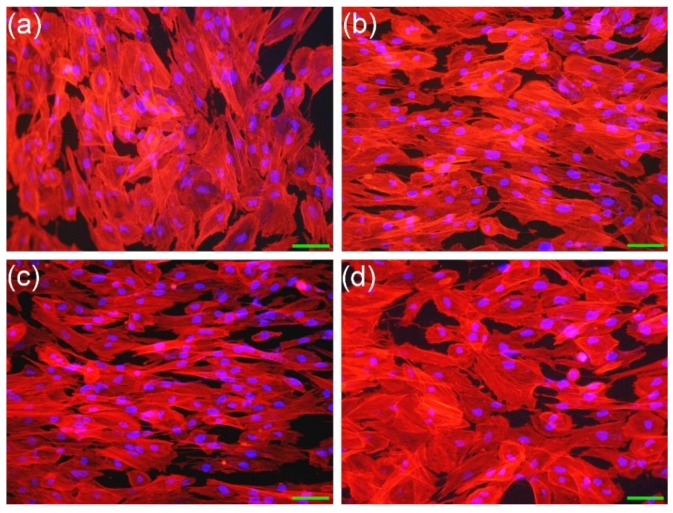
Morphology of fibroblasts (Hs27) grown on different substrates (**a**) bare 410SS; (**b**) 5A; (**c**) 10B; and (**d**) 20C films, respectively. The actin cytoskeleton was stained with phalloidin-AlexaFluor596 (red) and cell nuclei counterstained with DAPI (blue). Objective 10×. Magnification bar = 25 µm.

Cells presented a normal aspect with a polygonal or spindle shape, being well spread on the surface (Figure 2). The actin microfilaments of the cytoskeleton displayed a normal appearance in the case of all analyzed situations. As a side note, although the overall dimensions of the cells were roughly the same, one can observe that on the surface of 10B films (Figure 2c), the cells tended to have a more elongated (spindle) shape than in the case of the other types of surfaces (Figure 2a,b,d). Cell nuclei had a normal aspect and dimension in the case of all samples. The morphology testifies to the good adherence and biocompatibility of TiN thin films.

#### 3.2.2. Cell Proliferation

This was investigated by a classic MTS test. Due to the particularity of the experiment involving cells grown on opaque substrates, the classic protocol suggested by the manufacturer was adapted as presented in Section 2.2.1.3. We used the same concentration of reagent diluted in cell culture media and the amount of liquid transferred in the microplate (*i.e.*, 120 µL), in order to use values from calibration experiments to assess the number of cells.

**Figure 3:**
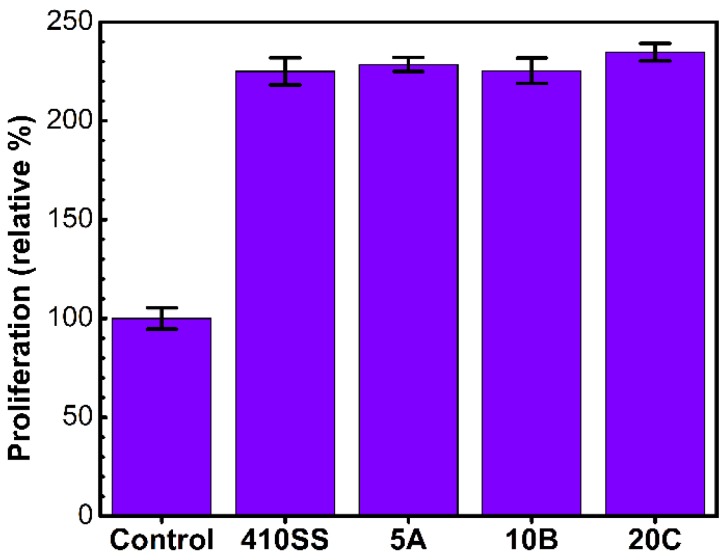
Histogram showing the cell proliferation results as obtained by the MTS assay. The values are normalized as the percent to the absorption of the seeding cell number.

Similar values of absorption were recorded for all samples. As presented in Figure 3, no significant differences were found between the four types of samples.

#### 3.2.3. Cell Death

LDH is an intracellular enzyme found in all cells. When a cell dies, it releases the active enzyme into the cell culture medium, and therefore, the LDH activity in the cell culture media is proportional to the number of dead cells.

**Figure 4:**
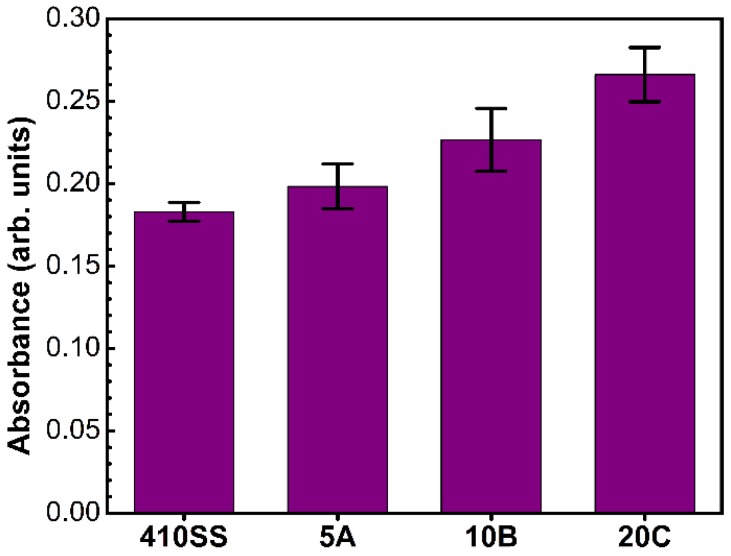
Cytotoxicity of samples: LDH assay. Values are in arbitrary absorption units showing the amount of dead cells in the case of the bare 410SS substrate, 5A, 10B and 20C films, respectively.

The cytotoxicity results (Figure 4) showed that the most biocompatible TiN film is 5A, which presented low values of cellular death, similar the 410SS substrate. The highest cytotoxicity was recorded for the 20C films, with an increase of ~45% with respect to the stainless steel control material (*p* < 0.05). All situations recorded an index of cell death lower than 3% of the total number.

However, there are some interesting aspects that deserve to be pointed out. Although we have seen that the 20C films determine a higher cellular death with up to 45% compared to the control, the overall cellular mortality is under 3%, the cell morphology and number after 24 h being very similar (with no significant differences) between the three types of TiN films. This peculiar situation can be the effect of a slightly poorer cell adhesion to the surface in the first 5 h. As will be shown by the XPS results, the surface that comes in direct contact with the cells has modified properties that arise from different concentrations of TiN in the surface and bulk volume of the films (Figure 9c). Since the mechanical properties of the three films are similar, based on the cytocompatibility studies, we mention that the 5A coatings should be considered a good candidate for the fabrication of surgical tools that come into contact with living tissues.

### 3.3. Physical-Chemical Investigations of TiN Films

The eye inspection of the TiN-deposited films revealed that the color of the film prepared in N_2_ atmosphere changed from light-yellow to gold-yellow, when increasing the number of applied laser pulses. In order to elucidate the difference in cell morphology and cytotoxicity exhibited by TiN films of different thicknesses, a series of physical-chemical investigations was conducted for identification of the possible factors that influence the biological response of films.

#### 3.3.1. Morphological Examination of TiN Films

At first, the focus was put on the films’ morphology, and we tried to identify significant topographic differences that could explain the cells’ affinity for the surfaces of the thinnest films.

**Figure 5:**
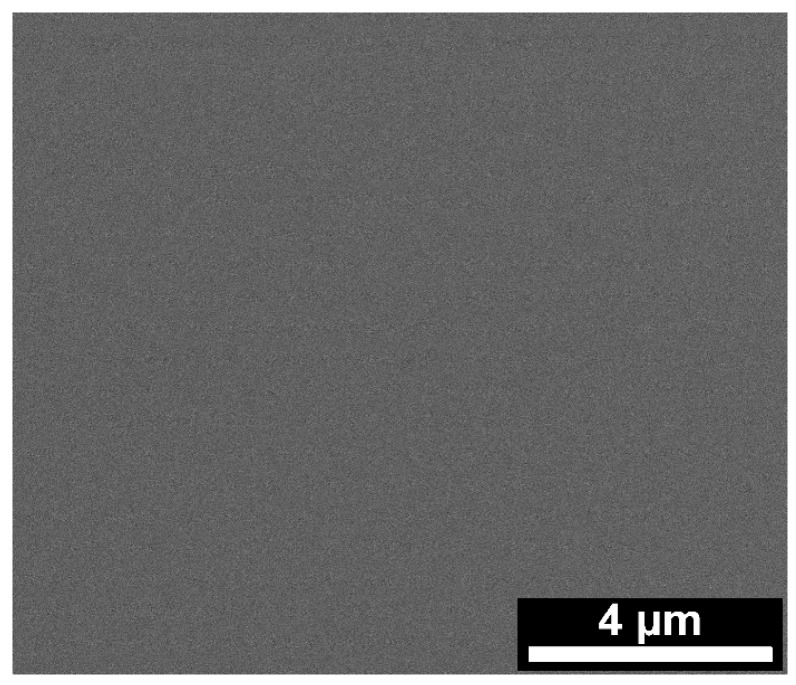
Typical SEM image of the 5A TiN film surface.

First, top-view SEM analyses were performed on the surface of the TiN films deposited by PLD on 410SS substrates. These investigations indicated that the PLD deposition regime chosen in our study led to films that present a homogeneous, smooth, continuous and pore-free microstructure, without particular morphological features. This is interesting, because, while indeed a large part of ablated matter is expulsed in gaseous state, some other (3%–5%) leaves the target in the form of “particulates” [28,39,40]. We therefore assume that the smooth nature of the films might be also explained by the high speed of the melted particles, which collapse and flatten when hitting the substrate and that are continually pressed by the next waves of incoming matter. None of the coatings showed evidence of micro-cracks or other defects and delamination. A typical SEM image of a TiN film is presented in Figure 5.

**Figure 6:**
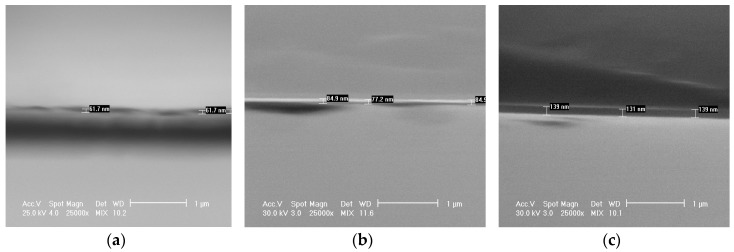
Typical cross-sectional SEM images of the TiN 5A (**a**), 10B (**b**) and 20C (**c**) films deposited onto mirror-polished Si wafers.

The cross-view SEM images (Figure 6) recorded for TiN films deposited on Si (100) wafers revealed the compact aspect of the PLD coatings, with similar thickness values as those determined by profilometry. We note that, in the case of thickness determination, performed for films deposited onto Si wafers, an interfacial layer of native oxide of 2–3 nm was taken into account. We emphasize also that the thickness of the TiN coatings was found uniform along the surface of the substrates.

**Figure 7:**
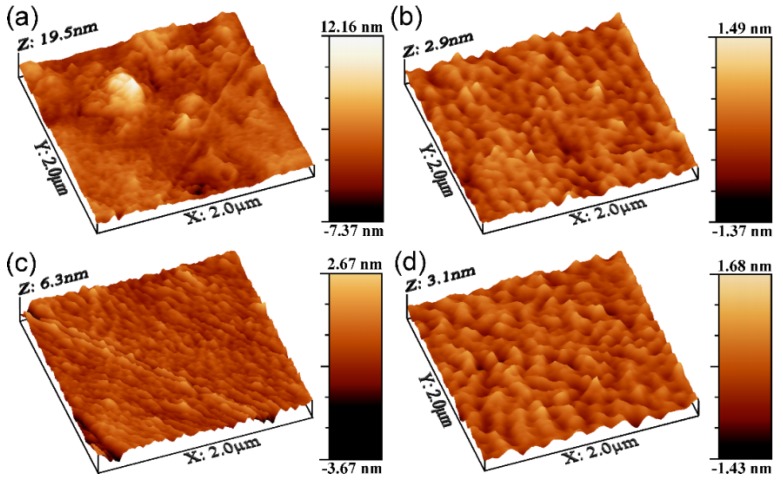
AFM images collected on 2 × 2 µm^2^ areas in the case of (**a**) bare glass substrate, (**b**) 5A, (**c**) 10B and (**d**) 20C TiN films.

Further, the AFM investigations have been performed in order to accommodate a more insightful analysis of the films topological features down to the nanometer scale, without further sample preparation. A higher contrast microscopy technique, such as AFM, was necessary to better discern the fine topological features of the TiN films. In Figure 7, representative 3D images for each investigated sample are given. The AFM topography of the deposited layers (Figure 7b–d) revealed the formation of quite smooth and uniform layers, with a morphology different from the one of the glass substrate (Table 3, Figure 7a). When scanning different areas, one finds similar roughness values, which confirm the films’ uniformity. The mean roughness values are summarized in Table 3 and presented as the mean of three consecutive measurements ±SD. It can be observed that, in comparison to the non-coated substrates (bare surfaces), the roughness of the layers drastically decreases (~6-times) with the increasing number of applied laser pulses. Even though the surface roughness of the initial substrate is low (Table 3), the values obtained for TiN coatings showed a slight improvement of surface finishing by leveling up the hills and valleys present on the non-coated substrate (Figure 7a). The 5A and 20C TiN structures had an apparent tendency to arrange better, resulting in smoother films. The morphology of sample 10B, depicted in the AFM image (Figure 7c), seems denser than the ones recorded for the 5A and 20C surfaces, but in fact, one can observe that the *z*-axis of 10B presents higher values. This is due to higher (taller) grains and, consequently, the deeper valleys between them. However, due to the low roughness values (Table 3), at the resolution limit of the apparatus, it is difficult to stress an evolutionary morphology trend, as even a small substrate defect could induce some degree of variation. Therefore, these results should be treated with caution.

**Table 3:** Roughness (*R*_RMS_ and *R*_a_) values of deposited TiN films determined by AFM on scanning areas of 2 × 2 µm^2^.

Sample Type	*R*_RMS_ (nm)	*R*_a_ (nm)
Reference (bare glass substrate)	1.76 ± 0.02	1.33 ± 0.03
5A film	0.30 ± 0.01	0.23 ± 0.01
10B film	0.60 ± 0.03	0.44 ± 0.02
20C film	0.37 ± 0.02	0.29 ± 0.02

### 3.4. Compositional Analyses of TiN Films

As the morphological investigations showed no significant differences between samples that could warrant a topographical hypothesis as the cause of the different biological behaviors, the next step was to conduct chemical surface analysis on all samples by XPS.

**Figure 8:**
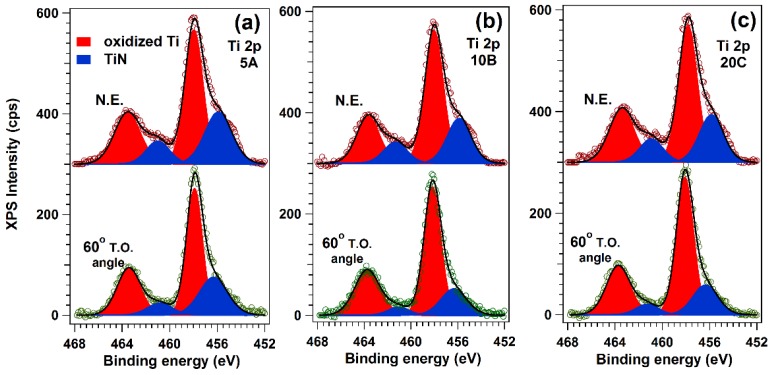
Ti2p XPS spectra recorded at N.E. with respect to the electron analyzer and at a 60° T.O. in order to increase the surface sensitivity, in the case of (**a**) 5A; (**b**) 10B and (**c**) 20C TiN films, respectively.

Ti2p XPS spectra (Figure 8) featured two contributions: one due to Ti atoms in the TiN environment, situated at lower binding energies (455.8–455.9 eV), and a second one, at higher binding energies (458 eV), associated with the presence of oxidized Ti (TiO_2_, TiN*_x_*O*_y_*) [41,42]. The binding energies of the oxidized species are close enough to each other, so it is reasonable to describe them as a single component. Therefore, the structure of the Ti2p spectrum suggests that the samples in fact feature a combination of TiN and Ti in the oxidized state. Based on the value of the inelastic mean free path of Ti photoelectrons at the corresponding kinetic energy, ΛTi = 1.9 nm, we estimate a probing depth of approximately 5.7 nm (~10 unit cells) in normal emission measurement and 2.8 nm in the surface-sensitive one. The surface-sensitive measurements indicated that there is a systematic TiN enrichment toward the surface of the samples.

**Figure 9:**
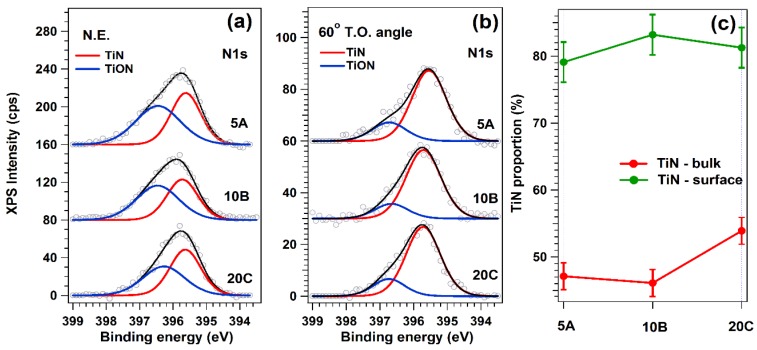
N1s XPS spectra of TiN films, recorded at (**a**) N.E. with respect to the electron analyzer and at (**b**) a 60° T.O. in order to increase the surface sensitivity; in (**c**) is depicted the amount of TiN in each sample for both bulk and surface-sensitive measurements.

N1s spectra (Figure 9) featured two components, one positioned at a binding energy of 395.5–395.6 eV, associated with N in TiN, and one centered at higher binding energies, associated with a TiN*_x_*O*_y_* compound [43]. They confirm the trend emphasized in the case of the Ti2p composition (Figure 8).

Based on the Ti2p and N1s integral amplitudes, corrected with their photoionization cross-sections, the experimental TiN stoichiometry has been deduced, and the results are presented in Table 4.

It is evident, on the one hand, that all samples are N-enriched, with the thinnest close to the ideal stoichiometry. On the other hand, the thicker ones are even more N-enriched at the surface. In the thickest sample, there is practically no difference between the bulk and surface stoichiometry (Figure 9a,b).

**Table 4:** Stoichiometry of TiN films as determined by XPS analyses.

Sample Type	Stoichiometry		
	5A	10B	20C
Volume	TiN_1.19_	TiN_1.27_	TiN_1.37_
Surface	TiN_1.10_	TiN_1.39_	TiN_1.36_

### 3.5. Structural Investigation of TiN Films

**Figure 10:**
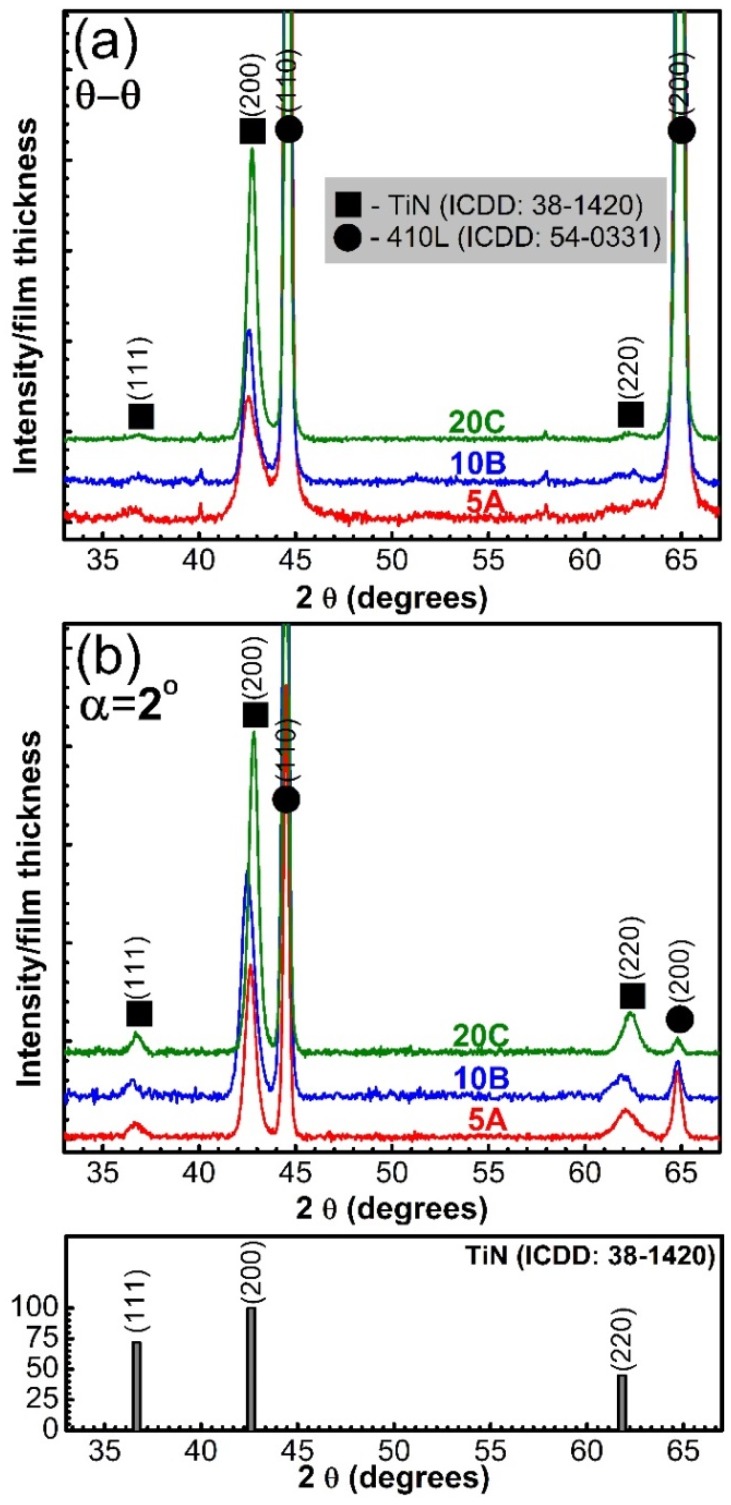
XRD patterns of the TiN thin films, recorded in (**a**) symmetric (θ–θ); and (**b**) grazing incidence (α = 2°) geometry. Bottom of the figure: ICDD reference file No. 00-038-1420 of cubic TiN.

The XRD diagrams collected in symmetric θ–θ geometry (Figure 10a) revealed, as expected, the prominent presence of the 410SS substrate peaks (ICDD: 00-054-0331). In order to discriminate the lower intensity diffraction peaks originating from deposited films, the intensity scales of the graphical representations were modified as appropriate.

The PLD thin films were polycrystalline, exhibiting reflections assigned to the TiN B1 cubic structure (ICDD: 00-038-1420). The patterns recorded in symmetric geometry indicated the predominance of the 200 TiN line (ICDD: 00-038-1420), with the other diffraction lines of TiN (111 and 200) as feeble, poorly-defined peaks. The differences in intensity ratios with respect to the reference file (bottom of Figure 10) point towards a 200 preferential growth of the TiN thin films.

The grazing incidence XRD patterns of TiN coatings are shown in Figure 10b. Using this measurement geometry, all of the TiN diffraction maxima were more clearly emphasized. The small fixed entrance angle causes the path traveled by the X-rays to increase significantly; thereby, thin film diffraction line intensities will be enhanced, making it also possible to evidence the crystal planes inclined with respect to the sample surface, whose normal will be the bisector of the angle formed by the incident and the diffracted beam. However, such a technique is not adequate to study preferred orientation tendencies, thereby the necessity of using both symmetrical and grazing incidence measurement geometries in this study.

The crystalline coherence length (“crystalline size”) of the TiN films was estimated using the integral breadth of 200 lines, by applying the Scherrer equation [44]. The lines width was corrected for instrumental broadening using a corundum standard reference (NIST SRM 1976). An enhancement of the crystallite size was detected when the number of applied laser pulses was increased: ~9.5 (5A), ~13.6 (10B) and 15.1 nm (20C), respectively.

### 3.6. Wetting Behavior of TiN Films

**Figure 11:**
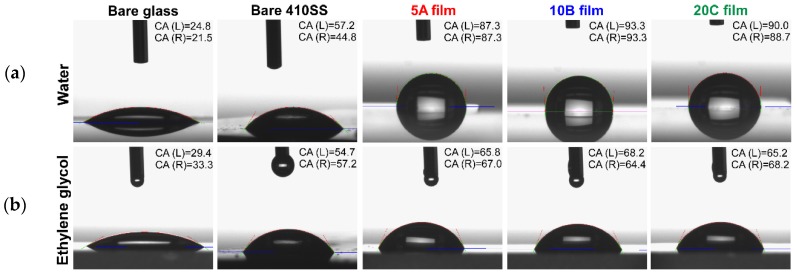
Typical contact angle images recoded for bare and TiN-coated substrates. (**a**) Water; (**b**) ethylene glycol.

Table 5 presents the variation of the surface CA with liquids of different polarity (*i.e.*, water and ethylene glycol). The values of SFE (total value and the values for each component) inferred from CAs are also shown. Figure 11 presents typical CA images recorded in the case of bare and TiN-coated specimens.

From the values collected in Table 5, one can observe differences in the wetting behavior in the case of reference substrates (glass and 410SS) when compared to deposited films. TiN coatings exhibited CA values of 1.8–4.0-times higher, when using water, and of 1.2–2.1-times higher, when using ethylene glycol as wetting solvents, with respect to the reference substrates. For the bare substrates, the CA values were less than 60°, while in the case of covered ones, they were of 90°. One can therefore conclude that the initial hydrophilic surfaces become hydrophobic, after TiN deposition. It is to be stressed that the CA values of deposited films differ by 2°–3° only between investigated areas on a given sample.

The nanostructuring of the deposited layers is higher than the one of the substrate, and the value of the apparent CA is bigger than the one of a similar smooth surface [45,46]. The gaps between grains are filled with air acting therefore as a support “buffer” for the water droplet. As a consequence, the contact to the surface is restricted to a few nanometric areas only.

The CA values obtained for all deposited films were close enough, and no significant differences could be observed between surfaces.

The SFE values for TiN-deposited films were lower than those of bare 410SS and glass substrates (Table 5). One can also notice an important decrease (31%–34%) of the SFE after applying the TiN coating: from 76.1 mN/m for the bare 410SS substrate down to 23.8–25.9 mN/m for the TiN/410SS-deposited structures. It is to be mentioned that the obtained SFE value of 76.1 mN/m in the case of glass substrate is close to the one reported in the literature [32,47].

**Table 5:** Contact angle and surface free energy values of the bare and deposited samples.

Sample Type	Contact Angle (°)		Surface Free Energy		
	Water	Ethylene Glycol	γ_s_^d^	γ_s_^p^	γ_total_
Bare glass	23.2 ± 0.4	31.4 ± 0.3	3.8	72.3	76.1
Bare 410SS	51.0 ± 1.9	56.0 ± 1.1	2.4	53.2	55.6
5A film	88.8 ± 2.12	66.4 ± 0.3	15.2	8.7	24.0
10B film	93.2 ± 0.07	66.3 ± 0.2	23.2	2.7	25.9
20C film	88.5 ± 1.27	66.7 ± 0.5	16.9	6.9	23.8

## 4. Discussion

We note that, during our studies, no differences between films deposited on various substrates were observed.

Biological tests on TiN films deposited by PLD in different conditions revealed a strong connection between their thickness and biological response. The main question to be answered was: “which is the factor that is thickness dependent and highly influential for biological response?”. The first hypothesis was to check the surface morphology. It is known that crystallite size increases with films thickness [48], and this could affect the overall structure, arrangement and, likely, the surface morphology of the films. However, microscopic investigations of the surface revealed defect-free and dense structures with roughness values less than 1 nm. Bollen, *et al.* [49,50] and Größner-Schreiber, *et al.* [51] showed that a surface roughness less than 200 nm has no effect on bacterial adhesion, because most of the bacteria have larger sizes, while Katsikogianni, *et al.* [52] and Tsang, *et al.* [53] showed that an increase in the surface roughness (superior to the aforementioned value) can encourage cell adhesion by creating more favorable sites for colonization inside surface irregularities [54]. Therefore, it is highly unlikely that the small differences in roughness between our TiN films of different thicknesses could affect the biological behavior of films. Consequently, our attention was focused on film structure and surface chemistry.

XRD investigations demonstrated the existence of B1-structure TiN films. These types of structures have been found to possess key properties for technological applications, such as: hardness, excellent adhesion, high strength and rigidity, wear-corrosion resistance, high thermal stability, low friction coefficient and good chemical stability [55]. Crystallite size increased with film thicknesses, being ~6 nm larger in the case of the 20C samples as compared to the 5A ones.

The adhesion strength at the coating-surface interface represents a critical parameter for the long-term stability of medical devices. The high values of adherence obtained in the case of TiN coatings should be therefore emphasized.

When a cell adheres and spreads, between the substrate surface and its membrane remains a nanometric space containing water molecules, ions, cell adhesion proteins and substrate-adsorbed proteins and organic substances. Therefore, the spreading of a cell on a surface is promoted by a sum of factors, like the hydrophilic behavior of the surface (SFE), the presence of some moieties, protein adsorption on the surface, surface roughness, texture, *etc*. In order to predict the cell adhesion and spreading, there is no simple answer, the outcome being determined by all of these factors that are partially linked to one another.

Cell spreading on a surface is a complicated and dynamic process that requires an equilibrium between tension and elastic forces on the membrane surface, cellular internal pressures, cytoskeleton rearrangement, molecule interactions, *etc*. In our case, the factors are: the polar component of the SFE and the surface composition (TiN*_x_*O*_y_* concentration and TiN stoichiometry).

Wettability is a critical factor that can directly influence the cells’ adhesion and growth. It is worth noting that in the case of our TiN samples, the water contact angles were between 80° and 90°. Tamada, *et al.* [56] and Chang, *et al.* [57] reported that angles around 80° provide maximum adhesion for fibroblast cell cultures. SFE results have shown that the polar free energy decreases in the case of deposited films as compared to bare substrates. This is probably due to the lack of polar groups on the TiN-finished surfaces, which determines a weakening of the bond strength connected to the polar elements of the liquid. The polar component of surface energy plays a key role in determining the hydrophobic behavior of the surface [58]. Therefore, in the case of deposited films, the higher the value obtained for CA, the lower is the value for the polar component of SFE. The particular growth of fibroblast cells on the surface of the 10B sample (more elongated compared to the polygonal aspect of the cells seeded onto the other two types of samples (5A and 20C)) could be related to the higher SFE value recorded in the case of this type of surface (26 *vs.* 24 mJ/m^2^).

The XPS analyses showed differences in terms of the surface and bulk composition of films, depending on their thickness. As the cells “see” only the surface and not the volume of the sample, the attention was mainly focused on the evolution of the surface composition. The films consisted of a mix of titanium nitride (with various stoichiometry) and titanium oxynitride (TiN*_x_*O*_y_*) (Figure 9c). Further, even though all films exhibit good cytocompatibility, a difference between the biological responses of samples was detected, depending on the TiN_x_O_y_ concentration and TiN stoichiometry. The samples with lower TiN*_x_*O*_y_* surface content induce more cellular death, ~45% with respect to the stainless steel control material (*p* < 0.05) *vs.* 12% in the case of a TiN*_x_*O*_y_*-rich surface. However, Banakh, *et al.* [59] showed that for a wide range of TiN*_x_*O*_y_* ratios, the structures remain biocompatible, without major viability and proliferation differences as a function of the atomic ratios of films. Therefore, it is suggested that in our case, the TiN surface stoichiometry is the key compositional factor that influences the cytotoxicity. The closer the films’ stoichiometry is to the theoretical one, the higher is the biocompatibility. This could be the reason why the thinnest sample (5A), having a Ti:N ratio of 1:1.1, is slightly more biocompatible than the other two (10B and 20C), which contain TiN compounds more enriched in nitrogen.

Our hypothesis is that the TiN stoichiometry variation with thickness (Table 4) comes from the difference in the atomic weight of titanium and nitrogen. Considering that TiN is ablated congruent from the target, the plasma plume will consist of an approximately equal amount of titanium and nitrogen. Because titanium is heavier, it tends to go deeper into the film, a process that is stimulated by the multi-pulse deposition leading to a higher thickness of the film. Nitrogen would have a tendency to accumulate closer to the surface of the film. When the films are thinner (e.g., 5A), these accumulation areas are very close to each other and even overlap. The reconstitution of TiN occurs under continuous substrate heating. In this case, a Ti/N ratio closer to unity is obtained. In the case of thicker films, there is a wider gap between titanium and nitrogen accumulation areas. Therefore, close to the surface, there are zones richer in nitrogen.

Even under high vacuum, there are still traces of oxygen in the residual gas [28,39,60]. The oxygen becomes more reactive with temperature, and the deposited layer always includes oxide impurities. The phenomenon is more visible in the case of thin films and stands at the origin of the formation of TiN*_x_*O*_y_* compounds (Figure 9c).

## 5. Conclusions

Complex physical-chemical, mechanical and biological studies have been carried out on titanium nitride (TiN) thin films of various thicknesses, synthesized by pulsed laser deposition in low nitrogen pressure in order to explain a better biocompatibility observed in the case of thinner films. By comparing the samples to the control glass substrate, a fibroblast mortality higher with 45% was observed in the case of films of ~130 nm as compared to 12% obtained in the case of ~60 nm-thick films. As all of the films were extremely smooth, with roughness values of under 1 nm and crystallites of comparable sizes, the only main difference between films was found in their surface composition. XPS data revealed that all films’ surfaces consisted of a mix of non-stoichiometric TiN and TiN*_x_*O*_y_*. The decisive factor for the biological response was found to be the TiN stoichiometry: the closer to unity, the more biocompatible the film is.

A thin TiN film (*i.e*., 5A ≈ 60 nm) should be considered as the optimum solution. This assumption is further sustained by experimental physical-chemical, mechanical and biological evidence and further allows for minimum production costs and reduced environmental pollution. These support the potential use of very thin TiN films for improving the characteristics of medical instruments (e.g., drills, burrs, scalpels or chisels), where sharpness and edge retention are important.
